# “Star of Bethlehem sign” in the analysis of the evolution of brain lesions during and after treatment for neuroparacoccidioidomycosis

**DOI:** 10.1590/0100-3984.2023.0030

**Published:** 2023

**Authors:** Larissa M. Santana, Paulo Mendes Peçanha, Aloísio Falqueto, Wdson L. M. Kruschewsky, Tânia Regina Grão-Velloso, Sarah Santos Gonçalves, Marcos Rosa-Júnior

**Affiliations:** 1 Hospital Universitário Cassiano Antônio Moraes da Universidade Federal do Espírito Santo (HUCAM/UFES/EBSERH), Vitória, ES, Brazil; 2 Universidade Federal do Espírito Santo (UFES), Vitória, ES, Brazil; 3 Hospital das Clínicas da Faculdade de Medicina da Universidade de São Paulo (HC-FMUSP), São Paulo, SP, Brazil; 4 Santi Medicina Diagnóstica, Vitória, ES, Brazil; 5 Hospital Meridional Vitória, Kora Saúde, Vitória, ES, Brazil

**Keywords:** Paracoccidioidomycosis, Central nervous system, Fungal infections, Tomography, X-ray computed, Magnetic resonance imaging, Invasive fungal infections, Paracoccidioidomicose, Sistema nervoso central, Infecções fúngicas, Tomografia computadorizada, Ressonância magnética, Infecções fúngicas invasivas

## Abstract

**Objective:**

To describe the clinical and radiological evolution of lesions during and after treatment in patients diagnosed with neuroparacoccidioidomycosis (NPCM).

**Materials and Methods:**

This was a retrospective study of the medical records, computed tomography scans, and magnetic resonance imaging (MRI) scans of patients with NPCM treated between September 2013 and January 2022.

**Results:**

Of 36 cases of NPCM, eight were included in the study. One patient presented only with pachymeningeal and skull involvement, and seven presented with pseudotumors in the brain. Collectively, the eight patients presented with 52 lesions, of which 46 (88.5%) were supratentorial. There were 32 lesions with a diameter ≤ 1.2 cm, of which 27 (84.4%) disappeared during the treatment. In three cases, there were lesions > 1.2 cm that showed a characteristic pattern of evolution on MRI: an eccentric gadolinium contrast-enhanced nodule, with a subsequent decreased in the size and degree of contrast enhancement of the lesions.

**Conclusion:**

In NPCM, supratentorial lesions seem to predominate. Lesions ≤ 1.2 cm tend to disappear completely during treatment. Lesions > 1.2 cm tend to present with a similar pattern, designated the “Star of Bethlehem sign”, throughout treatment.

## Abstract (pt)

**Objetivo:**

Descrever a evolução clínica e radiológica das lesões durante e após o tratamento de pacientes diagnosticados com neuroparacoccidioidomicose (NPCM).

**Materiais e Métodos:**

Revisamos os prontuários médicos, estudos de tomografia computadorizada e ressonância magnética (RM) de pacientes com NPCM de nossa instituição, no período de setembro de 2013 a janeiro de 2022.

**Resultados:**

Dos 36 casos de NPCM, oito foram incluídos no presente estudo. Um caso apresentava apenas envolvimento paquimeníngeo e ósseo craniano e sete casos apresentavam lesões encefálicas pseudotumorais, totalizando 52 lesões, sendo 46 (88,5%) supratentoriais. Dentre 32 lesões com diâmetro ≤ 1,2 cm, 27 (84,4%) apresentaram resolução completa durante o tratamento. Três casos apresentaram padrão semelhante de evolução da lesão na RM em lesões > 1,2 cm, caracterizado pelo aparecimento de nódulo excêntrico com impregnação pelo gadolínio, seguido de redução das dimensões e do realce nodular pelo contraste nos estudos subsequentes.

**Conclusão:**

A NPCM apresenta-se predominantemente com lesões supratentoriais. Lesões ≤ 1,2 cm tendem a desaparecer completamente durante o tratamento. Lesões > 1,2 cm tendem a apresentar um padrão de imagem de RM característico ao longo do tratamento, descrito como o “sinal da Estrela de Belém”.

## INTRODUCTION

Paracoccidioidomycosis (PCM) is a granulomatous systemic mycosis caused by thermodimorphic fungi of the genus *Paracoccidioides*^(1,2)^. There is no evidence of human-to-human transmission of PCM; infection is acquired by inhalation of conidia or propagules that reach the pulmonary alveoli and are transformed into yeasts capable of causing focal areas of pneumonitis. From those primary pulmonary foci, the fungus can spread, reaching the various organs and systems of the host by the lymphatic or hematogenous route^(3)^.

The geographical distribution of PCM is predominantly in Latin America, the disease being most common in the countries of Brazil, Colombia, Venezuela, Argentina, and Ecuador^(4,5)^. Central American countries, the Caribbean, and southern Mexico are considered to be of low endemicity^(6)^. To date, no cases have been reported in Chile, Nicaragua, Suriname, Belize, or Guyana. The estimated incidence in highly endemic areas is 1-4 cases/100,000 population per year^(7)^.

Case reports of PCM outside endemic areas are related to individuals who lived in or at least traveled to Latin America at some time in their lives, because the latency period of the disease ranges from days to years^(8,9)^. In a systematic review, Wagner et al.^(9)^ identified 83 cases of PCM diagnosed in Europe since 1985. Cases of PCM have been reported in 11 European countries: Austria, Bulgaria, France, Germany, Great Britain, Italy, Portugal, Spain, Switzerland, the Netherlands, and Norway. There have also been reports of cases in the United States, Canada, Japan, and Africa^(10)^.

The incidence of central nervous system (CNS) involvement in PCM, often referred to as neuroparacoccidioidomycosis (NPCM), varies across studies, ranging from 1% to 27%, and such involvement is more commonly observed in chronic cases, with mortality rates as high as 53%^(2,11-14)^.

In patients with NPCM, lesions in the CNS may present as pseudotumors (in approximately 90% of cases) or as meningeal (leptomeningeal or pachymeningeal) involvement (in approximately 10%). In such patients, the pseudotumors manifest as supratentorial or infratentorial intraparenchymal granulomas and can mimic neoplastic lesions or pyogenic abscesses^(14,15)^. The meningeal involvement manifests as inflammation of the pachymeninges or leptomeninges, usually at the base of the skull^(12,16)^. To our knowledge, there have been no studies evaluating the evolution of NPCM-related pseudotumors during treatment for the disease.

The objective of this article is to describe the clinical and radiological evolution of lesions during and after treatment in patients diagnosed with NPCM.

## MATERIALS AND METHODS

### Study design

This was a retrospective descriptive study of consecutive patients with a confirmed diagnosis of NPCM who were treated at the infectious disease unit of the Hospital Universitário Cassiano Antônio Moraes (HUCAM), in the city of Vitória, Brazil. Data from physical and digital hospital medical records were accessed, including images from computed tomography (CT) and magnetic resonance imaging (MRI) scans performed at the hospital between September 2013 and January 2022. The study was approved by the HUCAM Research Ethics Committee (Reference no. 68431717.3.0000.507).

### Data collection and inclusion criteria

All patients included in the study had a diagnosis of PCM, confirmed by direct mycological examination using 20% potassium hydroxide plus Parker ink or histopathological findings consistent with the disease, with or without serology for the exoantigens of *Paracoccidioides brasiliensis* (UFES-29 and B-339), performed by the double immunodiffusion method. We evaluated only patients presenting with at least one CNS lesion (i.e., patients with NPCM) and for whom at least two follow-up imaging studies (MRI, CT, or both) were available. The following variables were evaluated: age, sex, occupation, comorbidities, geographic location, treatment, and outcome. The criteria applied in order to define a cured case of NPCM were as follows: clinical improvement of symptoms; normalization of inflammatory markers (C-reactive protein and erythrocyte sedimentation rate); and negative serology for *P. brasiliensis*. Patients with pseudotumors in the CNS were treated until there were no more CNS lesions.

### Image analysis

All images were evaluated by two radiologists, with 2 and 11 years of experience, respectively, working independently with Digital Imaging and Communications in Medicine viewers (RadiAnt DICOM Viewer; https://www.radiantviewer.com). The images had been acquired in a 64-channel CT scanner (Toshiba Medical Systems, Tokyo, Japan) and a 1.5-T MRI scanner (Philips Healthcare, Best, the Netherlands). The characteristics of the CT and MRI scans were as previously reported by Rosa-Junior et al.^(14)^. No minimum or maximum interval between the initial and follow-up examinations was established. Lesions were measured, quantified, and described as to the type as well as to the pattern of contrast enhancement. The gadolinium-based contrast agent gadodiamide (0.1 mL/kg) was used in all MRI studies, and the nonionic, water-soluble contrast agent iohexol (1.0 mL/kg) was used in all CT studies.

### Data analysis

For all of the cases evaluated, epidemiological and clinical data were collected by using a standard clinical chart. Those data were entered into a Microsoft Excel spreadsheet and analyzed descriptively.

## RESULTS

During the study period, 844 patients were diagnosed with PCM at the HUCAM referral center for infectious diseases. Thirty-six (4.26%) of those patients were diagnosed with NPCM; eight met the study inclusion criteria and were analyzed in this investigation.

All eight patients included in the study were men, between 25 and 66 years of age (median, 50 years), who were residents of the Brazilian state of Espírito Santo. All eight presented with disseminated PCM, and only one was free of neurological symptoms at the time of diagnosis. All of the patients were smokers and consumed alcohol. None of them had tested positive for infection with HIV.

On evolutive serology, six patients (75%) tested negative for *P. brasiliensis* exoantigens during treatment, despite the findings of brain lesions. For two patients (25%), there were no serology results in the medical records. According to the results, all eight patients were infected with *Paracoccidioides* spp., and it was therefore not possible to discriminate the species. Seven of the patients had worked or were working as farmers, and one was in general services.

Five (62.5%) of the eight patients received amphotericin B as induction therapy, and all five were treated with trimethoprim-sulfamethoxazole thereafter. The duration of treatment ranged from 26 months to 108 months, and in two cases (25%), the treatment was irregular for a time. By January 2022, two patients (25%) had completed the treatment (after 13 and 75 months, respectively) and had evolved to a cure. Two other patients decided to discontinue treatment. The remaining four patients underwent quarterly follow-up examinations at the HUCAM referral center for infectious diseases. Table 1 summarizes the clinical and epidemiological characteristics of the cases.

**Table 1:** Demographic, clinical, and epidemiological characteristics of the patients with NPCM included in the study.

Case	Sex	Age (years)	Treatment	Comorbidities	Imaging follow-up (years)
1	Male	56	Amphotericin B and trimethoprim-sulfamethoxazole	Hypertension, smoking	0.5
2	Male	55	Amphotericin B and trimethoprim-sulfamethoxazole	Smoking, alcohol consumption	3.9
3	Male	38	Trimethoprim-sulfamethoxazole	Smoking	5.4
4	Male	64	Trimethoprim-sulfamethoxazole	Smoking, alcohol consumption	5.6
5	Male	45	Amphotericin B and trimethoprim-sulfamethoxazole	Smoking, alcohol consumption	7.6
6	Male	32	Trimethoprim-sulfamethoxazole	Hypertension, smoking and alcohol consumption	3.5
7	Male	66	Amphotericin B and trimethoprim-sulfamethoxazole	Smoking, alcohol consumption	3.0
8	Male	25	Amphotericin B and trimethoprim-sulfamethoxazole	Smoking	1.25

Of the eight patients evaluated, six (75%) presented at least four lesions for evaluation. A solitary CNS lesion was observed in only one case. We analyzed 52 nodular brain lesions, of which 46 (88.5%) were in the supratentorial compartment and six (11.5%) were in the infratentorial compartment. In the one remaining case (case 8), the patient had no lesions in the brain parenchyma, only involvement of the skull with extension to the adjacent pachymeninges. All the lesions showed contrast enhancement on imaging. The interval between the initial and final imaging studies varied between 0.5 and 7.6 years. There was no increase in lesion size and no formation of new lesions during the evaluation period. Of the 52 pseudotumors evaluated, 32 (61.5%) had a diameter ≤ 1.2 cm at the initial examination. Of those, 27 (84.4%) had disappeared by the time of the follow-up imaging studies, including 21 (87.5%) of the 24 pseudotumors with a diameter ≤ 0.8 cm at the initial examination (Table 2).

**Table 2:** Analysis of brain lesions in patients with NPCM.

Case	Infratentorial lesions (n)	Supratentorial lesions (n)	Lesions ≤ 0.8 cm		Lesions > 0.8 cm		All lesions (n)
			Total (n)	Disappeared (n)	Total (n)	Disappeared (n)	
1	0	5	1	0	4	0	5
2	0	5	0	0	5	0	5
3	5	15	15	15	5	4	20
4	0	1	0	0	1	0	1
5	0	4	2	1	2	0	4
6	1	5	5	5	1	1	6
7	0	11	1	0	10	1	11

**Figure 1:**
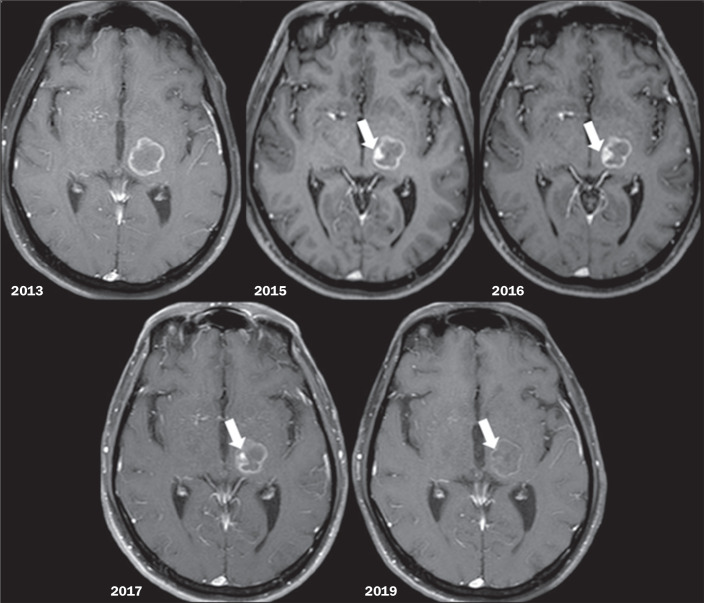
Axial contrast-enhanced T1-weighted images showing the appearance and progressive enlargement of the eccentric nodule (“Star of Bethlehem sign”, arrows), followed by a reduction in the size and degree of contrast enhancement of the nodule over time in a left thalamic lesion.

All eight cases presented some degree of vasogenic edema adjacent to the lesions at the time of the diagnosis. Of the seven cases presenting with cerebral pseudotumors, three presented three or more lesions on MRI scans. In those three cases (cases 2, 4, and 5), the MRI or CT scans were acquired at least one year after the initial scans. The imaging pattern observed during the treatment was similar in all three of those cases: an eccentric nodule with contrast enhancement on MRI in all lesions > 1.2 cm and subsequent progressive reduction of that nodule, a conjunction known as the “Star of Bethlehem sign”, although without a significant reduction in the overall size of the lesions (Figure 1).

In one of the cases evaluated in this study (case 1), the lesion remained stable in two consecutive follow-up imaging examinations, one of which was performed at six months after the end of treatment. In case 3, there were also only two follow-up imaging examinations, the second of which was performed more than five years after the initial examination. Of the 20 lesions initially identified in that case, 19 (all ≤ 1.2 cm) had disappeared by the time of the second follow-up examination. In the largest lesion, there was a reduction in size (from 1.2 cm to 0.4 cm) and in the degree of contrast enhancement. In case 6, there were six lesions (all ≤ 0.8 cm), which showed complete resolution at three months after the initial imaging study. At three years after the initial imaging study, nine of the ten of the lesions identified in case 7 showed a reduction in size, whereas the tenth lesion had disappeared, none having presented eccentric nodule enhancement during the follow-up studies.

**Figure 2:**
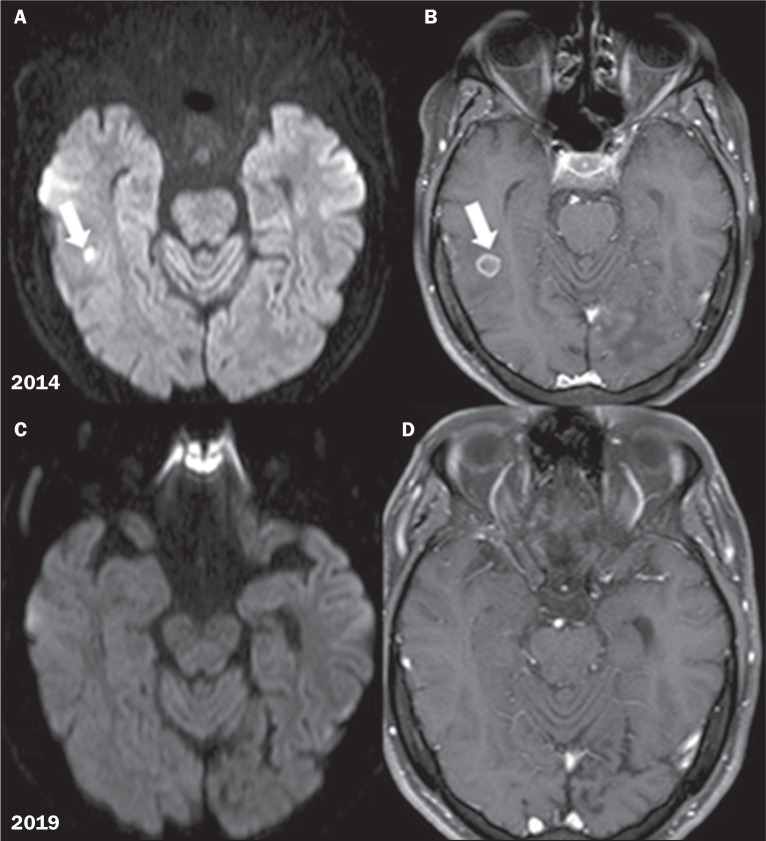
Diffusion-weighted imaging (DWI) analysis. A: Axial DWI showing central restricted diffusion in a small pseudotumor (arrow); B: Axial contrast-enhanced T1-weighted image showing peripheral enhancement (arrow); C,D: Follow-up imaging-axial DWI sequence and contrast-enhanced T1-weighted image, respectively-showing that the lesions had disappeared.

**Figure 3:**
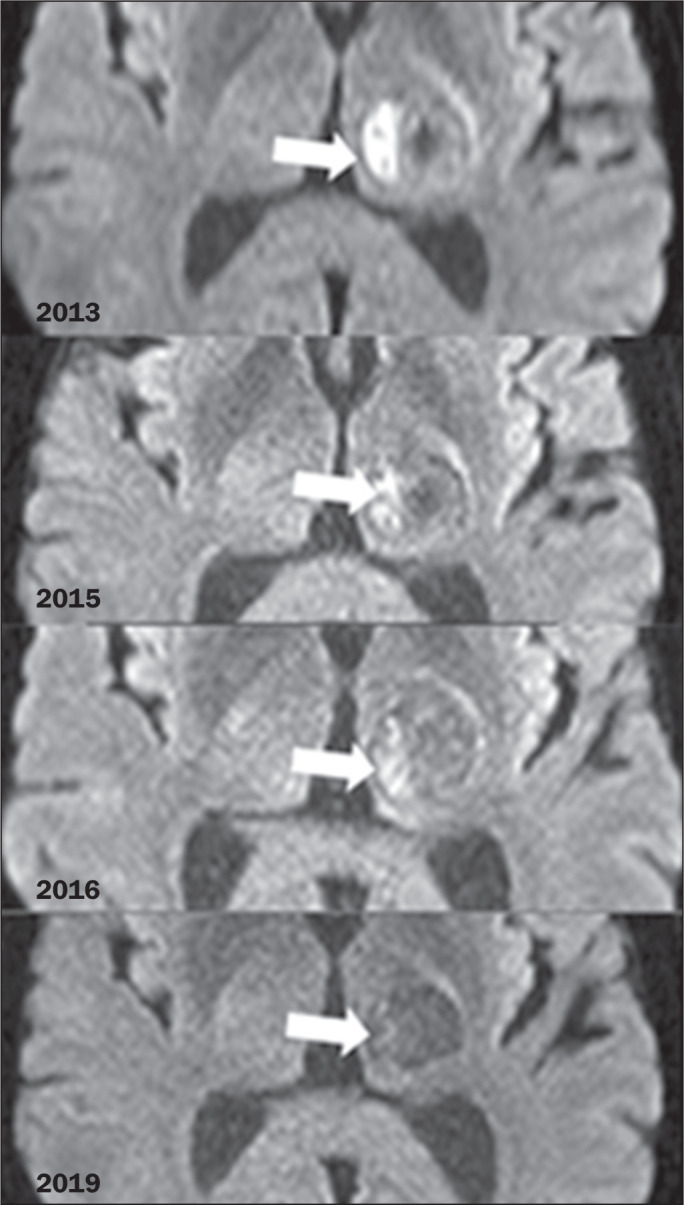
Axial DWI showing an area of peripheral restricted diffusion within the eccentric nodule (arrows) with a progressive reduction over time.

In the analysis of the diffusion-weighted MRI sequences, we observed central restricted diffusion in all of the lesions ≤ 1.2 cm. However, in the lesions > 1.2 cm, we observed peripheral restricted diffusion in some areas, with progressive reduction and disappearance of the restricted areas on follow-up scans. These findings are illustrated in Figures 2 and 3.

## DISCUSSION

The prevalence of NPCM varies widely in the literature, from 1.0% to 27.3%, and is higher in autopsy studies^(17-19)^. In the present study, the prevalence was 4.3%. However, we believe that prevalence was underestimated during the years of follow-up, given that some patients with CNS lesions are asymptomatic.

Alcohol consumption and smoking have been associated with the development of PCM^(20-22)^. A case-control study showed that smokers were 14 times more likely to develop the chronic form of PCM^(20)^.

The higher prevalence of NPCM in men and the predominance of lesions in the supratentorial compartment found in the present study are in agreement with the findings of other studies^(23,24)^.

The latest Brazilian consensus on PCM, published in 2017, establishes the need for imaging studies depending on the clinical presentation^(4)^. Although initially described as rare, probably due to asymptomatic presentations, NPCM has been diagnosed with increasing frequency because of the expansion of access to CT and MRI in recent decades^(2)^.

Conventional imaging studies of NPCM have specifically analyzed brain lesions, collecting data only on the patterns of distribution and characteristics of the lesions, mainly by CT or MRI^(2,14,15,24)^. Gasparetto et al.^(15)^ demonstrated a relationship between the time since symptom onset and the degree of enhancement of pseudotumors on CT, observing that hypodense lesions in patients evaluated within the first five months after symptom onset were hypodense, whereas those in patients evaluated more than five months after symptom onset were hyperdense. However, the authors did not provide an exact explanation for what that finding means.

In addition to being a radiation-free imaging modality, MRI is the best method for evaluation of the brain parenchyma and meninges, which should make it the imaging modality of choice for the evaluation of NPCM lesions^(12,14,18)^. Ring contrast enhancement on MRI has been described previously, as has the lipid peak during spectroscopy^(12,14)^. However, none of those conventional imaging studies have described the evolution of imaging aspects during treatment.

The appearance of the Star of Bethlehem, also known as the Christmas Star, was a celestial event that is believed to have occurred around the time of the birth of Jesus Christ. It is said to have been a bright, shining star that guided the three wise men to the birthplace of Jesus. Just as the Star of Bethlehem appeared to the three wise men, in the follow-up of patients undergoing treatment for NPCM, the “Star of Bethlehem sign”, characterized by an eccentric nodule showing contrast enhancement on MRI, appeared on T1-weighted images of all of the lesions > 1.2 cm, with a tendency to disappear during treatment. Such nodules were observed in three (43%) of the seven patients with pseudotumors. Although those nodules disappeared from within the lesions during treatment, the overall post-treatment decrease in the size of the large lesions was minimal after treatment. Those large lesions persisted for years after the end of treatment, raising the question of whether or not they were still active lesions during conventional imaging follow-up examinations.

In an abstract published at the 2018 Annual Congress of the European Association of Nuclear Medicine^(25)^, Cunha et al. described the use of ^18^F-fluorodeoxyglucose positron-emission tomography/CT for the evaluation of the extent of active disease in patients under treatment for PCM and demonstrated that it can be more sensitive than is conventional imaging for detecting active lesions.

The “Star of Bethlehem sign” in PCM is similar to the eccentric target sign described in patients with cerebral toxoplasmosis, the difference being that in toxoplasmosis, the sign appears in the initial examination rather than in follow-up examinations. Although the pathological correlation of this sign in cerebral toxoplasmosis is speculative, it is believed, as described by Kumar et al.^(26)^, that “the central enhancing core of the target seen on MRI [is] produced by a leash of inflamed vessels extending down the length of the sulcus that [is] surrounded by concentric zones of necrosis and a wall composed of histiocytes and proliferating blood vessels, with impaired permeability producing the peripheral enhancing rim.” The histopathological correlations of this finding in NPCM still need to be studied in order to clarify these questions.

Luthra et al.^(27)^ observed that, on MRI, fungal abscesses with intracavitary projections were not enhanced by gadolinium, whereas pyogenic abscesses presented with smooth or discretely lobulated margins. Similarly, all of the pseudotumors analyzed in the present study had irregular margins with intracavitary projections. We observed restricted diffusion in the parietal projections of fungal abscesses in the lesions > 1.2 cm. However, the pattern of central restricted diffusion that we observed in the lesions < 1.2 cm, described by Luthra et al.^(27)^ as characteristic of pyogenic and tuberculous abscesses, should not rule out the possibility of NPCM.

There have been various studies of the systemic and CNS changes in patients with PCM^(2,10-12,14-19,24,25,28-30)^. However, to our knowledge, this is the first conventional imaging study analyzing the behavior of NPCM lesions after treatment, as well as the largest imaging follow-up study of patients with NPCM. Nevertheless, our study has some limitations. The number of patients with documented CNS lesions was small. In addition, the interval between the initial and follow-up imaging studies varied widely because many of the patients were from rural regions that were far from the treatment center.

## CONCLUSION

In patients with NPCM, supratentorial pseudotumors seem to be the most common lesion type. Lesions ≤ 0.8 cm tend to disappear completely after treatment. Lesions > 1.2 cm tend to evolve with an eccentric enhancing nodule on contrast-enhanced T1-weighted images, with subsequent progressive reduction of this eccentric nodule and enhancement (the “Star of Bethlehem sign”), but without a significant reduction in the overall size of the lesions, even after years of treatment.
